# Cytotoxicity and proinflammatory effects of titanium and zirconia particles

**DOI:** 10.1186/s40729-019-0178-2

**Published:** 2019-07-09

**Authors:** Frank Schwarz, Maike Langer, Tina Hagena, Brigitte Hartig, Robert Sader, Jürgen Becker

**Affiliations:** 1 Department of Oral Surgery and Implantology, Carolinum, Goethe University, Frankfurt, Germany; 2 Department of Oral Surgery, Universitätsklinikum Düsseldorf, Düsseldorf, Germany; 3 Department for Oral, Cranio-Maxillofacial and Facial Plastic Surgery, Medical Center of the Goethe University Frankfurt, Frankfurt am Main, Germany

**Keywords:** Cellular immunology, Cytokines, Fibroblast, In vitro model, Monocytes, Osteoblast

## Abstract

**Background:**

To assess the effects of differently sized titanium (Ti) and zirconia (Zr) particles on (1) the metabolic activity of osteosarcoma-derived osteoblasts (SaOs-2) and human gingival fibroblasts (HGF) and (2) the cytokine expression of monocytes (THP-1)

**Methods:**

Ti (60–80 nm and 100 nm) and Zr (2 μm and 75 μm) particles were incubated with SaOs-2, HGF, and THP-1 cells. At days 0, 2, 4, and 7 and 0, 1, 2, and 4 (THP-1), the mitochondrial activity was assessed and enzyme-linked immunosorbent assays were used to determine interleukin (IL)-1 beta and IL-6 concentrations of stimulated THP-1 at day 1.

**Results:**

Ti60–80, Ti100, Zr2, and Zr75 particles were associated with gradual and significant within-group decreases in the viability of SaOs-2 and HGF cells. These effects were less pronounced in the Zr group. Similar to control cells, THP-1 did not reveal any significant increases in IL-1 beta and IL-6 concentrations. Viability of THP-1 was merely impaired in the presence of Ti100.

**Conclusions:**

Ti and Zr particles had a detrimental effect on the viability of SaOs-2 and HGF, but no proinflammatory effect on THP-1.

## Background

Recently, peri-implantitis was defined as “a plaque-associated pathological condition occurring in tissues around dental implants, characterized by inflammation in the peri-implant mucosa and subsequent progressive loss of supporting bone.” [1]. It was also anticipated that titanium (Ti) and metal particles may contribute to the pathogeneses of peri-implant disease [2, 3]. These particles can be released either by abrasion during implant insertion [4], micromovements at the implant-abutment interface [5, 6], or biocorrosion [7, 8]. In fact, histological analyses of human biopsy material obtained at peri-implantitis sites suggested an association between the inflammatory cell infiltrate and detectable metal particles [9, 10]. However, metal-like debris was also noted at healthy implant sites [11], thus questioning the role of titanium or metal particles in the pathogenesis of peri-implant diseases [12]. Nevertheless, several in vitro studies provide some evidence that microsized and nanosized titanium particles may induce cytotoxic effects [13–16] and enhance pro-inflammatory responses [17–20].

Very limited data also suggest that particle release from implants made of yttria-stabilized tetragonal zirconia polycrystal (Zr) may induce similar cellular reactions [21, 22]. Since these implants reveal similar biological complications [23] as Ti implants, one may also have to question the potential role of Zr particles in the pathogenesis of peri-implant disease. A major limitation of currently available studies on both Ti and Zr particles is the lack of cell lineages with a major relevance to peri-implant tissues.

Therefore, the aim of the present study was to assess and compare the effects of differently sized Ti and Zr particles on (1) the viability of osteoblasts and fibroblasts and (2) the cytokine expression of monocytes in vitro.

## Methods

### Titanium and zirconia particles

Commercially pure (99.9%) Ti particles exhibiting average particle sizes of either 60–80 nm (Ti60–80) or 100 nm (Ti100) as well as Zr particles of 2 μm (Zr2) and 75 μm (Zr75) (io-li-tec, Heilbronn, Germany) were used for the present analysis.

### Cell cultures

Binding 96-well plates (Corning Inc., Acton, MA, USA) containing Ti60–80, Ti100, Zr2, and Zr75 particles (applied to homogeneously cover the well ground in a monolayer) as well as empty controls were seeded with human gingival fibroblasts (HGF; Provitro GmbH, Berlin, Germany) (6 wells per group, in duplicate), human osteogenic osteosarcoma cells (SaOs-2, DSMZ, Braunschweig, Germany) [3000 cells] (6 wells per group, in triplicate), and acute monocytic leukemia cells (THP-1, DSMZ, Braunschweig, Germany) [5000 cells] (5 wells per group) and cultured in 1 ml of Dulbecco’s modified Eagle’s medium (DMEM; high glucose, GlutaMax, Sigma-Aldrich, Schnelldorf, Germany) with the supplement of 10% fetal bovine serum (FBS; Sigma-Aldrich) and 1% penicillin/streptomycin.

One group of THP-1 cells was stimulated using phorbol 12-myristate 13-acetate (5 ng/ml) (PMA; P8139-1MG, Merck KGaA, Darmstadt, Germany) and served as an additional control.

The cell culture conditions were set at a temperature of 37 °C and a humidified atmosphere of 95% and 5% CO_2_.

### Cell viability assay

Cell viability was measured at days 0, 2, 4, and 7 (HGF and SaOs-2) and days 0, 1, 2, and 4 (THP-1) by the use of a luminescence assay (CellTiter-Glo®; Promega, Mannheim, Germany) in a luminometer (Victor X3; Perkin-Elmer, Rodgau, Germany). This assay is based on a quantification of adenosinetriphosphate (ATP), signaling the presence of metabolic active cells. This is based on the luciferase-catalyzed reaction of luciferin and ATP. In particular, mono-oxygenation of luciferin is catalyzed by luciferase in the presence of Mg^2+^, ATP, and molecular oxygen. The luminescent signal was measured in counts per second (CPS).

### Enzyme-linked immunosorbent assays

Enzyme-linked immunosorbent assays (ELISA) were used to determine human interleukin (IL)-1 beta and IL-6 (Quantikine® ELISA kit, R&D Systems Inc., Minneapolis, USA) concentrations at 24 h following incubation of Ti60–80, Ti100, Zr2, and ZR75 particles with PMA (5 ng/ml)-stimulated THP-1 cells (DSMZ, Braunschweig, Germany) [5000 cells; in duplicate, binding 24-well plates with the same culture conditions as described above]. THP-1 seeded in empty wells served as cell controls. Recombinant human cytokines (Quantikine® Immunoassay Control Group 1, R&D Systems Inc.) were used as additional positive controls (IL-1 beta, 40–79 pg/ml; IL-6, 80–98 pg/ml; TNF-alpha, 147–265 pg/ml).

Standard curves (*x*-axis: IL-1 beta/IL-6/TNF-alpha concentrations in pg/ml; *y*-axis: optical densities) were established according to the instructions given by the manufacturer (Quantikine® ELISA kit). Optical densities of each well were assessed using a microplate reader (Victor X3; Perkin-Elmer, Rodgau, Germany) at 450 nm.

### Statistical analysis

A software package (SPSS 24.0, SPSS Inc., Chicago, IL, USA) was used for the statistical analysis. Mean values, standard deviations, medians, min., and max. were calculated for each group. Analysis of variance (ANOVA) and post hoc testing using Bonferroni’s correction for multiple comparisons was used for within-group comparisons of cell viability measurements. Comparisons of mean optical densities were accomplished using the unpaired *t* test. Results were considered statistically significant at *P* < 0.05.

## Results

**Fig. 1:**
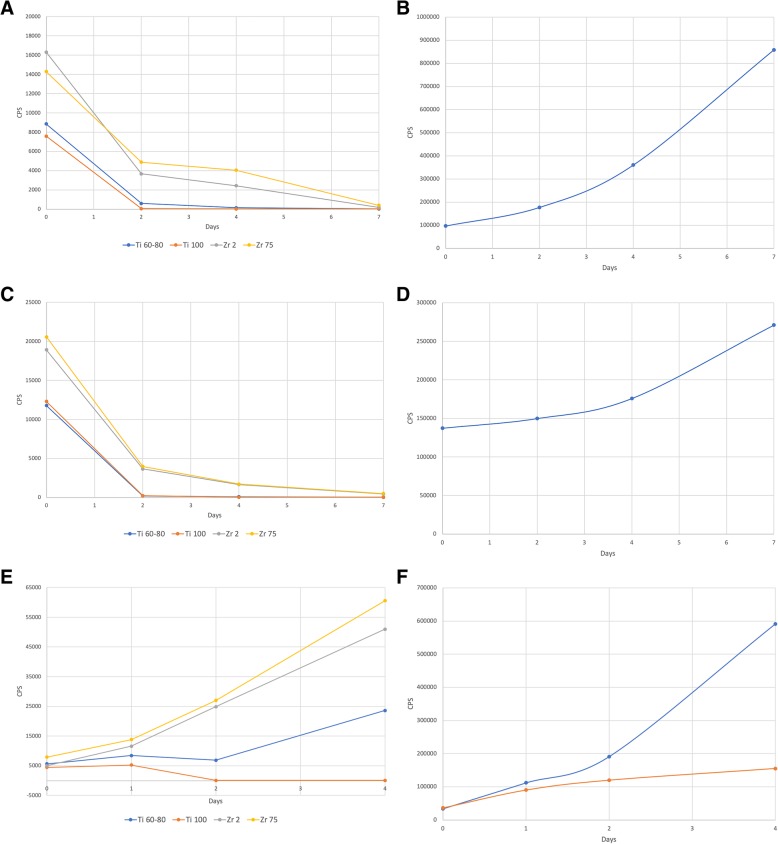
Mitochondrial activity expressed as median luminescent output (counts per second -CPS) of SaOs-2 and HGF in different groups. **a** Test groups (SaOs-2). **b** Control group (SaOs-2). **c** Test groups (HGF). **d** Control group (HGF). **e** Test groups (THP-1). **f** Control groups (THP-1)

The viability of SaOs-2 and HGF cells in test and control groups expressed as luminescent output (median CPS) is presented in Figs. 1a–d.

### Cell viability assay SaOs-2

In all test groups investigated, within-group comparisons revealed significant decreases of mean CPS values at days 2, 4, and 7 (Fig. 1a).

In particular, CPS in the Ti60–80 group decreased from 8109.28 ± 3361.91 (min. 4439.0, max. 11039.5) at day 0 to 672.9 ± 248.05 (min. 481.0, max. 953.0) at day 2 (*P* = 0.004), to 113.44 ± 43.54 (min. 63.3, max. 142.0) at day 4 (*P* = 0.002), and to 27.66 ± 13.32 (min. 17.3, max. 42.7) at day 7 (*P* = 0.002), respectively. In the Ti100 group, CPS decreased from 7907.67 ± 2723.49 (min. 5360.0, max. 10778.0) at day 0 to 87.54 ± 69.51 (min. 40.0, max. 167.0) at day 2 (*P* = 0.001), to 30.11 ± 9.29 (min. 23.0, max. 41.0) at day 4 (*P* = 0.001), and to 26.44 ± 8.86 (min. 16.0, max. 33.0) at day 7 (*P* = 0.001), respectively.

Similar reductions were also noted in both Zr groups. In particular, CPS in the Zr2 group decreased from 14,368.1 ± 6165.94 (min. 7463.0, max. 19324.0) at day 0 to 4141.89 ± 937.53 (min. 3539.0, max. 5222.0) at day 2 (*P* = 0.025), to 1981.22 ± 1013.28 (min. 821.0, max. 2690.0) at day 4 (*P* = 0.008), and to 245.34 ± 139.78 (min. 140.0, max. 404.0) at day 7 (*P* = 0.004), respectively. In the Zr75 group, CPS decreased from 15,072.0 ± 2914.94 (min. 12620.0, max. 18295.0) at day 0 to 4532.88 ± 2137.64 (min. 2244.0, max. 6478.0) at day 2 (*P* = 0.001), to 3192.78 ± 1700.42 (min. 1235.0, max. 4305.0) at day 4 (*P* = 0.001), and to 425.80 ± 84.72 (min. 366.0, max. 523.0) at day 7 (*P* = 0.001), respectively.

In contrast, the control wells were associated with significant (i.e., days 4 and 7) increases of mean CPS values from 100,043.20 ± 15,695.83 (min. 86363.0, max. 117179.0) at day 0 to 174,172.40 ± 31,103.18 (min. 141704.0, max. 203702.0) at day 2 (*P* = 1.00), to 346,257.23 ± 27,639.35 (min. 314427.0, max. 364188.0) at day 4 (*P* = 0.046), and to 828,144.28 ± 164,532.45 (min. 650621.0, max. 975516.0) at day 7 (*P* = 0.001), respectively (Fig. 1b).

### Cell viability assay HGF

When compared with day 0, all particles investigated were associated with significant within-group decreases of mean CPS values at days 2, 4, and 7 (Fig. 1c).

In particular, CPS in the Ti60–80 group decreased from 11,785.25 ± 2311.46 (min. 10150.8, max. 13419.7) at day 0 to 202.85 ± 139.79 (min. 104.0. max. 301.7) at day 2 (*P* = 0.003), to 82.00 ± 44.26 (min. 50.7, max. 113.3) at day 4 (*P* = 0.003), and to 34.35 ± 14.63 (min. 24.0, max. 44.7) at day 7 (*P* = 0.003), respectively. In the Ti100 group, CPS decreased from 12,303.55 ± 3716.90 (min. 9675.0, max. 14932.0) at day 0 to 209.35 ± 109.81 (min. 132.0, max. 287.0) at day 2 (*P* = 0.017), to 29.65 ± 8.41 (min. 24.0, max. 36.0) at day 4 (*P* = 0.016), and to 26.50 ± 8.76 (min. 20.0, max. 33.0) at day 7 (*P* = 0.016), respectively.

Mean CPS decreases were lower in both Zr groups. In particular, CPS in the Zr2 group decreased from 18,923.80 ± 10,591.04 (min. 11435.0, max. 26413.0) at day 0 to 3658.50 ± 64.34 (min. 3613.0, max. 3704.0) at day 2 (*P* = 0.272), to 1644.35 ± 761.76 (min. 1106.0, max. 2183.0) at day 4 (*P* = 0.188), and to 439.95 ± 189.01 (min. 306.0, max. 574.0) at day 7 (*P* = 0.152), respectively. In the Zr75 group, CPS decreased from 20,572.0 ± 6662.64 (min. 15861.0, max. 25283.0) at day 0 to 3968.50 ± 2585.46 (min. 2140.0, max. 5797.0) at day 2 (*P* = 0.059), to 1711.00 ± 542.63 (min. 1327.0, max. 2095.0) at day 4 (*P* = 0.037), and to 499.30 ± 161.22 (min. 385.0, max. 613.0) at day 7 (*P* = 0.030), respectively.

In contrast, the control wells were associated with increases of mean CPS values from 137,319.90 ± 16,984.13 (min. 125310.0, max. 149330.0) at day 0 to 149,859.90 ± 14,552.11 (min. 139570.0, max. 160150.0) at day 2 (*P* = 1.000), to 175,868.30 ± 12,545.48 (min. 166997.0, max. 184739.0) at day 4 (*P* = 1.000), and to 271,069.35 ± 76,582.98 (min. 216917.0, max. 325222.0) at day 7 (*P* = 0.178), respectively (Fig. 1d).

### Cell viability assay THF-1

When compared with day 0, Ti60–80, Zr2, and Zr75 particles were associated with increases of mean CPS values, reaching statistical significance at days 2 (Zr2 and Zr75) and 4 (Ti60–80, Zr2, and Zr75), respectively. In particular, mean CPS values in the Ti60–80 group increased from 5640.40 ± 741.96 (min. 4364.0, max. 6206.0) at day 0 to 8435.60 ± 2850.84 (min. 3478.0, max. 10460.0) at day 1 (*P* = 1.000). At day 2, mean CPS values decreased to 6845.60 ± 6332.99 (min. 36.0, max. 13362.0) (*P* = 1.000), but further increased to 23,615.40 ± 7678.70 (min. 14789.0, max. 34061.0) at day 4 (*P* < 0.001), respectively.

A more steady and pronounced increase in cell viability was noted in both Zr2/Zr75 groups, resulting in changes of mean CPS values from 5014.80 ± 989.10 (min. 3786.66, max. 6242.94)/7880.00 ± 892.51 (min. 6771.79, max. 8988.21) at day 0 to 50,980.60 ± 10,504.14 (min. 37937.98, max. 64023.22)/60,586.60 ± 3306.45 (min. 56481.10, max. 64692.10) at day 4 (*P* < 0.001), respectively (Fig. 1e).

In contrast, after a slight initial increase from 4439.20 ± 1571.87 (min. 2487.46, max. 6390.94) at day 0 to 5259.60 ± 5984.03 (min. − 2170.56, max. 12689.76) at day 1 (*P* = 1.000), Ti100 particles were associated with marked decreases of mean CPS values at days 2 (52.80 ± 15.65, min. 33.36, max. 72.24) (*P* = 0.237) and 4 (84.80 ± 106.94, min. − 47.99, max. 217.59) (*P* = 0.245), respectively.

In both control groups (THP-1 with and without PMA stimulation), within-group comparisons revealed significant increases of mean CPS values at days 1, 2, and 4 (*P* < 0.001), respectively. However, these increases in THP-1 viability were markedly lower following PMA stimulation (Fig. 1f).

### IL-1 beta and IL-6 ELISA

The optical density of IL-1 beta measurements was 0.047 ± 0.002 (median 0.046) in the Ti60–80 group, 0.046 ± 0.001 (median 0.047) in the Ti100 group, 0.047 ± 0.004 (median 0.046) in the Zr2 group, and 0.046 ± 0.003 (median 0.046) in the Zr75 group, respectively. These values were comparable to those noted in the respective control group (0.053 ± 0.006, median 0.053) (*P* > 0.05, respectively). Measurements of positive controls amounted to 0.43 ± 0.006 (median 0.43).

The optical density of IL-6 measurements was 0.040 ± 0.002 (median 0.040) in the Ti60–80 group, 0.038 ± 0.038 (median 0.038) in the Ti100 group, 0.040 ± 0.005 (median 0.04) in the Zr2 group, and 0.044 ± 0.001 (median 0.044) in the Zr75 group, respectively. These values were comparable to those noted in the respective control group (0.043 ± 0.001, median 0.043) (*P* > 0.05, respectively). Measurements of positive controls amounted to 0.37 ± 0.006 (median 0.37).

## Discussion

The present study was designed to investigate the effects of differently sized Ti and Zr particles on the viability of SaOs-2 osteoblasts and HGFs as well as the viability and cytokine expression of THP-1.

When compared with the respective control groups, it was observed that all particles investigated had a detrimental effect on the viability of SaOs-2 and HGF cells, as evidenced by significant reductions in mean CPS values. In contrast, the viability of THP-1 was merely impaired in the presence of Ti100. In this context, it must be emphasized that the luminescent signal, generated during cell lysis, is proportional to the amount of ATP present and therefore directly correlates with the number of viable cells [24].

To the best of our knowledge, these are the first in vitro analyses employing two cell lineages with a major relevance to peri-implant tissues (i.e., gingival fibroblasts, osteoblasts).

Basically, however, the present viability assessment corroborates previous analyses on the cytotoxicity of Ti particles [13–16]. In particular, Choi et al. [16] also reported on a significant decrease of the viability of osteoblasts at 72 h following incubation with differently sized Ti particles (i.e., < 1.5, ≥ 1.5, and < 5.0 μm; ≥ 5.0 and < 10.0 μm; and ≥ 10.0 and < 15.0 μm). Moreover, it was noted that particles of < 1.5 μm and up to 5.0 μm were clearly identifiable in the cytoplasm, whereas larger particle sizes were attached to the plasma membrane [16]. In this context, it must be emphasized that SaOs-2 cells as employed in the present analysis were characterized as osteoblast-like cells [25, 26], responding in a similar way to implant surfaces as primary human osteoblasts [27].

Comparable outcomes were also noted for a human pulmonary endothelial cell line and THP-1, also revealing that larger Ti particles had a more intense and negative effect on cell viability at 24 h than smaller particles (596 vs. 166 nm) [13].

Similarly, nanosized (20–250 nm) Ti particles were associated with a higher uptake and cytotoxic effects in periodontal ligament fibroblasts than microsized (0.3–43 μm) Ti particles [14]. This was also confirmed by Cai et al. [15] pointing to the highest cytotoxic effect of Ti nanoparticles that were within the range of 100 nm.

When further analyzing the present data, it was also observed the CPS reductions in HGF were commonly less pronounced when exposed to Zr particles, as compared with both Ti groups.

In this context, it must be emphasized that a major limitation of the present analysis was the lack of nanoscaled Zr particles. These particles, however, had also been obtained for this analysis, but due to electrostatic phenomena, their transfer to the well plates could not be accomplished. Nevertheless, the aforementioned data on the cytotoxic influence of different particle sizes may at least in part explain the higher mean CPS values in Zr2 and Zr75 groups.

On the contrary, however, the results of an experimental animal study provide some evidence that Ti microparticles were associated with a higher increase in O2− generation in macrophages than Zr particles, suggesting that their biocompatibility may have also been influenced by the “shape and/or crystal structure” of the metal itself [22].

The present analysis of cytokine expressions in THP-1 failed to corroborate recent findings on an enhanced proinflammatory response following exposure of THP-1 or macrophages to Ti particles [17–20].

In particular, Taira et al. [20] observed a high-level expression of IL-1 beta, IL-6, and TNF-alpha in THP-1 at 24 h. Similar responses were also noted in macrophages [17, 19]. When interpreting these discrepancies, one has to realize that a monocyte-like cell line, derived from acute monocytic leukemia cells (i.e., THP-1), may behave differently from primary macrophages [28]. Moreover, in peri-implant granulation tissue fibroblasts, an increase in the gene expression of IL-6, IL-8, and TNF-alpha was just measured when Ti particles were applied in sub-toxic doses [18]. Since none of the particles investigated in the present study were shown to impair the viability of stimulated THP-1 cells at day 1, one may assume that the absence of a cytokine expression was not caused by any cytotoxic effects.

Within its limitations, the present in vitro analysis revealed that Ti and Zr particles had a detrimental effect on the viability of SaOs-2 and HGF, but no proinflammatory effect on THP-1. These findings need to be validated in vivo.

## Data Availability

The availability of raw data used and/or analyzed during the current study is limited/restricted by general data protection regulations.
